# Influence of Traditional Cardiovascular Risk Factors on Carotid and Femoral Atherosclerotic Plaque Volume as Measured by Three-Dimensional Ultrasound

**DOI:** 10.3390/jcm8010032

**Published:** 2018-12-31

**Authors:** Maria Noflatscher, Michael Schreinlechner, Philip Sommer, Julia Kerschbaum, Katharina Berggren, Markus Theurl, Rudolf Kirchmair, Peter Marschang

**Affiliations:** 1 Department of Internal Medicine III (Cardiology, Angiology), Medical University of Innsbruck, Anichstr. 35, A-6020 Innsbruck, Austria; michael.schreinlechner@tirol-kliniken.at (M.S.); philip.sommer@i-med.ac.at (P.S.); katharina.berggren@gmx.de (K.B.); markus.theurl@i-med.ac.at (M.T.); Rudolf.Kirchmair@i-med.ac.at (R.K.); Peter.Marschang@i-med.ac.at (P.M.); 2 Department of Internal Medicine IV (Nephrology and Hypertension), Medical University of Innsbruck, Anichstr. 35, A-6020 Innsbruck, Austria; julia.kerschbaum@tirol-kliniken.at; 3 Department of Internal Medicine, Clinical centre of Traunstein, D-83278 Traunstein, Germany; 4 Department of Internal Medicine, Central Hospital of Bolzano, I-39100 Bolzano, Italy

**Keywords:** cardiovascular risk factors, atherosclerosis, cardiovascular clinical research, ultrasound

## Abstract

Background: Atherosclerosis is a systemic multifocal disease with a preference for the branching points of the arteries. In this study, we quantitatively measured carotid and femoral plaque volume in subjects with cardiovascular risk factors (CVRF) and/or established atherosclerotic disease using a 3D ultrasound technique. Methods: In this prospective, single-centre study, we included 404 patients (median age 64; 56.9% men) with at least one CVRF or established cardiovascular disease. Plaque volume was measured using 3D ultrasound equipped with an automated software. Results: We found a strong correlation of plaque volume with CVRF and the number of vascular beds involved. The strongest associations with total and femoral plaque volume were noted for smoking, hypertension, age, as well as for the presence of peripheral arterial occlusive disease (*p* < 0.05). Carotid plaque volume was best predicted by hyperlipidaemia, hypertension, age, as well as the presence of cerebrovascular disease and coronary artery disease (*p* < 0.05). Conclusion: We conclude that smoking appears to be associated with total and femoral plaque volume, whereas hyperlipidaemia seems to be associated with carotid plaque volume. Measurement of 3D plaque volume is a practical and reproducible technique with the potential to become an additional screening tool in cardiovascular risk stratification.

## 1. Introduction

Cardiovascular diseases are the leading cause of death worldwide [1]. Atherosclerosis is a systemic multifocal disease with a preference for the branching points of the arteries. Of all major vascular beds, only a few arteries, such as the carotid and femoral arteries, are easily accessible for non-invasive examinations using ultrasound probes. Ultrasound has several advantages compared to other non-invasive techniques, including the absence of radiation and contrast medium (compared to computed tomography), as well as low cost and broad availability (compared to magnetic resonance tomography) [2]. The carotid arteries have been analysed intensively with ultrasound for early signs of incipient arteriosclerosis including increased intima media thickness (IMT) and advanced plaque formation. The best examined parameter is the IMT, which can be easily measured using high frequency ultrasound probes, and has been suggested as a predictor for future cardiovascular events [3,4]. However, a recent meta-analysis raised doubt about the role of IMT as a predictor for cardiovascular events [5]. Consequently, the 2013 American College of Cardiology/American Heart Association (ACC/AHA) guidelines [6] do not recommend carotid IMT for routine risk assessments in patients without prior cardiovascular disease. Therefore, the detection and progression of atherosclerotic plaques may be a better predictor for future cardiovascular risk [7,8,9]. Although carotid plaques have been measured semi-quantitatively using different scoring systems, the precise measurement of the total plaque volume still remains a difficult task [10,11,12]. Belcaro et al. showed, in a large study with over 13,000 subjects followed for up to 10 years, that besides the carotid arteries, IMT and plaque detection in the femoral bifurcation is a significant predictor for cardiovascular events [13].

In the past, manually-performed, two- (2D) and three-dimensional (3D) methods for the quantifications of plaque volume have been used [14,15]. In patients undergoing coronary angiography, the 3D-system has a higher negative predictive value and sensitivity for the detection of concomitant coronary artery diseases (CAD) compared to 2D-ultrasonography [16]. Sonographic 3D plaque volumetry using specially-developed software is a promising new approach to precisely quantify atherosclerotic plaque volume in peripheral arteries. Sillesen and colleagues were the first to describe a piece of automated software for precisely quantifying plaques tested in the high-risk plaque BioImage study [17]. In this study, carotid plaque burden correlated better with coronary calcium measured by computed tomography than IMT, ankle brachial index, or abdominal aortic diameter.

Traditional risk factors for premature cardiovascular diseases like arterial hypertension, smoking, diabetes, hyperlipidaemia and family history are well known [18]. In recent decades, numerous inflammatory biomarkers including high-sensitivity C-reactive protein (hsCRP) and scoring systems such as the Framingham Risk Score (FRS) have become important for the risk stratification of individuals [6,19]. The recently-published PESA study showed an association between cardiovascular risk factors (CVRF) and plaque volume, especially in femoral arteries, using 3D-vascular ultrasound [20]. So far, the influence of CVRF on quantitatively-measured carotid and femoral plaque volume in subjects with CVRF and/or established atherosclerotic disease has not been systematically assessed. Therefore, our goal was to investigate the association of carotid and femoral plaque volume with traditional CVRF using this innovative 3D ultrasound approach.

## 2. Methods

This is a prospective observational single-centre cohort study (Correlation of Artherosclerotic Plaque Volume and Intima Media Thickness with Soluble P-selectin (ClinicalTrials.gov Identifier: NCT01895725)). To be eligible, patients between 30 and 85 years of age had to have an established cardiovascular disease (coronary artery disease (CAD), cerebrovascular disease (CVD), peripheral arterial occlusive disease (PAD)) or at least one traditional CVRF (arterial hypertension, smoking, hyperlipidaemia, diabetes, family history of cardiovascular disease). Family history of cardiovascular disease was defined as the occurrence of a premature cardiovascular event (myocardial infarction, stroke, or critical limb ischemia) in a first-degree relative (<55 years for male and <65 years for female relatives). The estimated glomerular filtration rate (eGFR) was calculated by the Modification of Diet in Renal Disease (MDRD) formula, and chronic kidney disease (CKD) was defined as estimated glomerular filtration rate (eGFR) <60 mL/min/1.73 m².

Between June 2013 and January 2018, all patients referred to the outpatient clinic at the Department of Internal Medicine III (cardiology, angiology) of Innsbruck Medical University for ultrasound examinations of the carotid and/or femoral arteries for standard indications were screened for potential inclusion into the study All subjects gave informed consent for inclusion before they participated in the study. The study was conducted in accordance with the Declaration of Helsinki, and the protocol was approved by the Ethics Committee of Innsbruck (Project identification code-UN5048).

At baseline, a sonographic examination with automated measurement of the IMT with a linear L9-3 probe and quantification of the plaque volume with a VL13-5 3D probe was performed using the Philips iU22 ultrasound system (Philips, Amsterdam, The Netherlands).

For each patient, a detailed history including cardiovascular disease and risk factors, coexisting diseases and current medication, as well as smoking status was recorded at the baseline visit.

In addition, all participants underwent an ankle brachial index and pulse wave velocity determination measured by an automated system (AngE Pro 4, SOT Medical Systems, Maria Rain, Austria).

At the same time, routine baseline laboratory analyses including total cholesterol, high density lipoprotein (HDL), low density lipoprotein (LDL), triglycerides, hsCRP, fasting glucose, Hemoglobin A1c, creatinine and eGFR were obtained. In addition, an EDTA blood sample was drawn from peripheral venous blood. The EDTA blood sample was centrifuged at 1730 g and the plasma was stored at −80 °C.

The FRS was calculated for each participant and classified accordingly in low (<10%), intermediate (>10–20%) and high (>20%) 10–years cardiovascular risk [21].

### 2.1. Ultrasound Imaging

**Figure 1:**
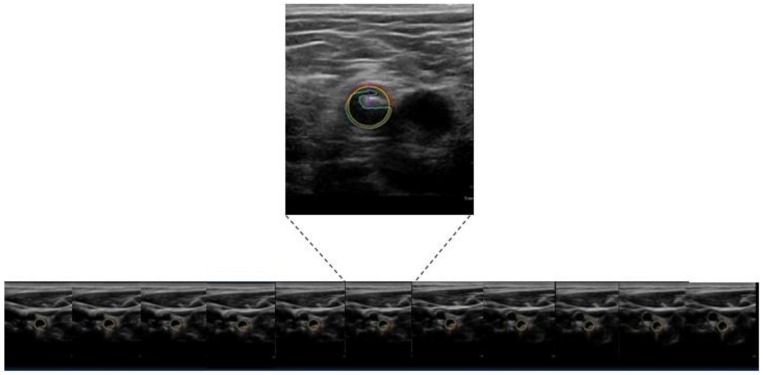
3D-measurement of plaque volume using images obtained from 3D ultrasound and plaque quantification software.

Each participant underwent a routine sonographic examination with flow velocity measurement of the common carotid artery, internal carotid artery, external carotid artery and vertebral artery as well as the common femoral artery, proximal superficial femoral artery and deep femoral artery. In addition, we performed an electrocardiogram-triggered measurement of the IMT following the recommendations of the Mannheim consensus [22] in the far wall of the distal common carotid artery, as well as the proximal superficial femoral artery 1 cm distal to the flow divider along a segment of 10 mm free of plaques. For the IMT measurements, we used a Philips iU22 system (Philips, Amsterdam, The Netherlands) equipped with a linear L9-3 probe using built-in, automatic mean IMT calculation software; measurements were performed in end-diastole (as determined by the R wave). Plaque volumetry was defined as local structure extending at least 0.5 mm into the arterial lumen, or 50% of the surrounding IMT, or showing a thickness >1.5mm, as measured from the media-adventitia interface to the intima-lumen interface. Plaque volumetry was performed using the Philips iU22 ultrasound system equipped (Philips, Amsterdam, The Netherlands) with a VL13-5 3D probe and plaque quantification software to assess the plaque volume on both sides. Plaque volume was measured for a distance of 6 cm within the bifurcation, and the adjacent parts of the internal and common carotid arteries, as well as within the common femoral artery, the femoral bifurcation and the adjacent parts of the proximal superficial femoral artery (Figure 1).

### 2.2. Statistical Analysis

The Kolmogorov and Smirnov Test was used to test for deviations from normal distribution [23]. Baseline data for continuous variables are presented as mean ± standard deviation (SD) for normally distributed parameters and as median and interquartile range (IQR) for parameters not following a normal distribution. Categorical variables are expressed as absolute numbers and percentages. The Mann-Whitney U test and the Kruskal Wallis test were used to evaluate differences of a continuous variable by a categorical independent variable with two or more groups, respectively. To compare categorical variables, the Chi square test was used.

The relationship between CVRF and total, femoral and carotid plaque volume was analysed by binary logistic regression. For this purpose, plaque volume was divided into high and low atherosclerotic plaque volume using the median of the distribution as a cut-off value (255.5 mm³ for total plaque volume, 139.5 mm³ for femoral plaque volume, and 79.5 mm³ for carotid plaque volume, respectively). First, the association of risk factors with the extent of plaque volume was evaluated by univariate logistic regression (data not shown). The variables in the multivariable model were smoking, hypertension, hyperlipidaemia, diabetes, family history of cardiovascular disease, age, sex, body mass index (BMI), peripheral arterial occlusive disease (PAD), coronary artery disease (CAD), cerebrovascular disease (CVD) and chronic kidney disease (CKD).

Generally, a *p*-value of <0.05 was considered as significant except when multiple testing was present. We then used the Bonferroni correction for multiple comparisons. Statistical analyses were conducted with SPSS Statistic (version 24.0; IBM Corp, Armonk, NY, USA).

## 3. Results

Here, we report the baseline data of the study designed to test the correlation of atherosclerotic plaque volume and intima media thickness with soluble P-selectin (ClinicalTrials.gov Identifier: NCT01895725). Due to slower enrolment than estimated, only 404 of the 600 originally planned patients with at least one CVRF or established cardiovascular disease were included. The median age of the overall population was 64 years (IQR: 56–71). Baseline characteristics, including demographic, clinical and laboratory characteristics, are summarized in Table 1.

Overall, our study participants belonged mainly to a low to intermediate risk population with a median FRS of 12.8% (Table 1) (IQR: 7.5–21.6%) and a percentage of 37.7% in the low risk group, 30.4% in the intermediate and only 29.5% in the high risk FRS group. In our study population, 122 patients suffered from CAD, 39 from CVD, and from 32 PAD. Notably, some patients had two or more cardiovascular beds involved. In particular, 11 participants had CAD and PAD, 11 had CAD and CVD and four had PAD and CVD. Six patients had all vascular beds involved.

All 404 participants received 3D volumetric ultrasonography. Inter-observer variability of three different observers revealed good agreement between the raters with an intra-class correlation coefficient of 0.95 (95% CI, 0.82–0.99).

Regarding the CVRF, most of the participants suffered from hypertension (65.3%), and even more from hyperlipidaemia (87.6%), with slightly more than half of the study population receiving antihypertensive (59.1%) and lipid lowering therapy (58.1%). There was a relatively low number of patients suffering from diabetes (12.6%), and nearly a quarter of the patients were current smokers and had a positive family history for cardiovascular diseases.

We divided the study population according to the total (carotid and femoral) plaque volume into two categories (low (0–255 mm³) and high (256–2048 mm³) total plaque volume), as shown in Table 1. Subjects with high total plaque volume were significantly older and more likely to be male compared to the group with low plaque volume. In addition, high total plaque volume was significantly associated with an increased number of subjects with hypertension, smokers (number of pack years), a higher FRS, higher hs-CRP, triglycerides and creatinine values, as well as lipid-lowering and antihypertensive therapy. Conversely, subjects with high total plaque volume were significantly less likely to have a family history of cardiovascular disease, high total LDL and HDL cholesterol values. No significant differences were observed for BMI, hyperlipidaemia, diabetes mellitus and antidiabetic therapy.

Table 2 shows the association of vascular disease with total plaque volume. Of our study population, 38% suffered from vascular disease, while 30% had CAD, nearly 10% had CVD, and 8% PAD. Patients with known vascular disease were more likely to have high total plaque volume, which was also significant for CAD and PAD. In contrast, individuals without vascular disease were significantly common in the group with high plaque volume.

In Table 3, the measured parameters (total, femoral and carotid plaque volume, carotid and femoral IMT, ankle-brachial index and pulse wave velocity) are shown for patients with low (0–255 mm³) and high (256–2048 mm³) total plaque volume, respectively. The median total plaque volume of our study population was 255.5 mm³ (IQR: 83–514 mm³), the median carotid plaque volume measured 79.5 mm³ (IQR: 12.3–240.3 mm³) and the median femoral plaque volume was 139.5 mm³ (IQR: 28.3–284 mm³). The ankle-brachial index was significantly lower, whereas the pulse wave velocity, the carotid IMT, and femoral IMT were significantly higher in patients with high total plaque volume compared to those with low total plaque volume.

### 3.1. Influence of the Number of Risk Factors on the Extent of Plaque Volume

Depending on the number of traditional CVRFs (arterial hypertension, smoking, hyperlipidaemia, diabetes, family history of cardiovascular disease), we observed a significant increase in total, femoral and carotid plaque volume (*p* < 0.001) (Table 4).

The majority of the participants (187, 47%) in our study had two CVRF with a median of 257 mm³ total plaque volume (IQR: 83–506 mm³) followed by one CVRF (123, 31%) with a median of 152 mm³ total plaque volume (IQR: 58–360 mm³), as shown in Table 4. Eighty-eight participants (22%) had ≥ three CVRF. In two patients with CVD, no evident CVRF could be identified. When comparing participants with two versus three or more CVRF, total plaque volume (257 mm³ (IQR: 83–506 mm³) vs. 448 mm³ (IQR: 167–701 mm³)), femoral plaque volume (144 mm³ (IQR: 29–290 mm³) vs. 215 mm³ (IQR: 84–445 mm³) and carotid plaque volume (87 mm³ (IQR: 11–222 mm³) vs. 139 mm³ (IQR: 52–351 mm³)) were consistently higher in patients with three and more CVRF compared to those with two CVRF.

### 3.2. Distribution of Atherosclerotic Plaque Volume According to Cardiovascular Disease

**Figure 2:**
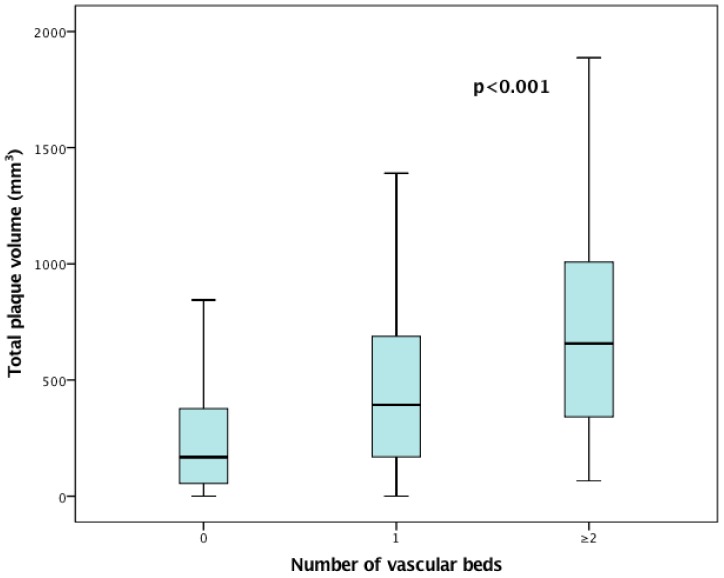
Association of total plaque volume with the number of vascular beds involved. The boxes show the median and interquartile range while the whiskers are representative of the 95% of the confidence interval.

We found that in the presence of cardiovascular disease (CAD, CVD and PAD), the amount of total plaque volume increased significantly compared to subjects without cardiovascular diseases. In fact, total plaque volume in patients with cardiovascular disease was more than twice as high compared to the group without cardiovascular disease (median of 442 mm³ (IQR: 197–796 mm³) versus 168 mm³ (IQR: 55–379 mm³) of total plaque volume. There was also a significant trend (*p* < 0.001) for increased plaque volume with increasing number of vascular beds involved (median of 393 mm³ (IQR: 166–691 mm³) for 1 vascular bed vs. median of 657 mm³ (IQR: 339–1036 mm³) for ≥2 vascular beds (Figure 2).

### 3.3. Relationship of Smoking Expressed in Pack-Years and Atherosclerotic Plaque Volume

Depending on smoking as expressed by the number of pack-years, the total and femoral plaque volume increased significantly. There was no significant change (*p* = 0.109) in carotid plaque volume in smokers vs. non-smokers (Table 5).

### 3.4. Association of CVRF, Cardiovascular Diseases and Plaque Volume

A multivariate prediction model for total, femoral and carotid plaque volume is shown in Table 6.

The strongest associations with total plaque volume were noted for smoking, hypertension and age, as well as for the presence of PAD and CAD, all of which were statistically significant. Hyperlipidaemia, diabetes, family history of cardiovascular disease, as well as the presence of CVD and CKD did not show a significant association with total plaque volume.

A similar association of CVRF and cardiovascular diseases was observed for femoral plaque volume with smoking, hypertension, age and the presence of PAD, but not CAD, as statistically-significant predictors.

Conversely, carotid plaque volume was best predicted by hyperlipidaemia, hypertension, age, as well as the presence of CVD and CAD with statistically significant associations. On the other hand, smoking, diabetes, PAD and CKD were not significant predictors for carotid plaque volume in our cohort. A family history for cardiovascular disease appeared to be associated with less carotid plaque volume.

## 4. Discussion

To our knowledge, this is the first study investigating total, femoral and carotid atherosclerotic plaque volume detected with 3D-sonography in patients with CVRF as well as in patients with established cardiovascular disease. We found a significant association of plaque volume with the number of CVRF and, in the subgroup of patients with cardiovascular disease, of vascular beds involved. Moreover, we were able to identify the individual risk factors predictive of total, femoral, and carotid plaque volume.

This study demonstrates that plaque volume determination using a commercially-available 3D-sonography system is a practicable and reproducible technique for the exact detection of subclinical atherosclerotic plaque burden. The BioImage-study, which was performed with a similar approach using a manual sweep with 2D-ultrasound probes followed by 3D-reconstruction, provided the first evidence of the value of plaque volume measurement in a large patient cohort [17]. The main merit of the 3D ultrasound system used in our study lies in the accurate measurement of the 3D structure of plaques, which is guaranteed by the automatic scanning process. Moreover, the built-in software makes it possible to determine the plaque volume within a reasonable time. In contrast, the price of the additional ultrasound probes as well as the additional time for the 3D examination has to be taken into consideration. When we divided our cohort into patients with high and low total plaque volumes, some (age, sex, hypertension, smoking, hs-CRP, HDL cholesterol, and triglycerides) but not all (BMI, hyperlipidaemia, diabetes mellitus) risk factors were significantly associated with plaque formation. Interestingly, a family history of cardiovascular disease was less frequently found in participants with high plaque volumes. Also in the PESA study, the authors did not observe an association of family history with plaque volume [20]. Moreover, elevated total and LDL cholesterol values were less frequently observed in participants with high plaque volumes in our study. This finding may be due to lipid-lowering therapy that was more often prescribed for individuals in the high plaque group.

CVRF have been associated with femoral and carotid plaque volume in two previous studies [24,25]. The Aragon Workers’ Heart Study [25] demonstrated a stronger correlation between risk factors and subclinical atherosclerosis in the femoral compared to the carotid arteries. However, in this study, the quantification of atherosclerosis was determined solely by the presence of plaques without exact quantification. Similarly, in the study by Yerli et al. [24], the measurement of atherosclerosis in the femoral arteries reflected better the exposure to CVRF than measurements in the carotid arteries. In the latter study, plaque quantification was performed using a linear 2D-ultrasound system measuring the 2D-plaque thickness and the plaque area.

Only one recently-published study, the PESA study, used the same 3D-vascular ultrasound technique for the quantification of the plaque volume, showing an association between CVRF and plaque burden, especially in femoral arteries [20]. However, the characteristics of the patients in the PESA study differed in several ways from our study. One main difference to our study is that the PESA-study included only participants without prior cardiovascular diseases. Secondly, the patients in the PESA study were considerably younger (mean age 46 years, compared to 64 years in our study), had fewer risk factors and belonged mostly (nearly 80%, compared to 38% in our study) to the low risk group. Accordingly, the mean total plaque volume was by far smaller (50.8 mm^3^) in the PESA study compared to our study (median total plaque volume 255.5 mm^3^).

To identify the individual contribution of risk factors, a multivariate analysis was performed. The only CVRF for all three parameters (total, femoral and carotid plaque volume) were hypertension and age. Smoking and PAD were strong predictors for total and femoral plaque volume, whereas hyperlipidaemia and CVD where strong predictors for carotid plaque volume. Our observations of the individual role of risk factors with plaque development in different vascular beds are in agreement with well-known clinical observations. As in the current study, the association of smoking was stronger for femoral and total plaque volume than for the carotid territory while hypertension showed no significant territorial differences in the PESA study [20]. While the CVRF hyperlipidaemia showed a stronger association with carotid than with femoral plaque volume in our study, the opposite was described in the PESA study [20]. Our findings are supported by a previous study of 1934 acute ischemic stroke patients which determined the relationship between total serum cholesterol and triglycerides and the grade of internal carotid artery stenosis [26]. This retrospective study described total cholesterol as an independent risk factor for atherosclerosis in the carotid artery. However, in this study the grade of stenosis and not plaque volume was used as quantification of atherosclerosis.

We expanded the findings of the PESA study by including not only patients with risk factors, but also with established cardiovascular diseases. As mentioned above, depending on the number of vascular beds involved (CAD, CVD and PAD), the burden of total plaque volume increased significantly (*p* < 0.001). Previously, two studies reported a strong correlation between peripheral arteriosclerotic plaque volume and coronary artery calcium [25,27]. These findings suggest that the non-invasive measurement of peripheral plaque volume may be a surrogate for atherosclerotic burden of the coronary arteries, and that screening for multisite arterial disease may be indicated in patients with CAD.

### 4.1. Strengths

The strength of our study is the determination of plaque volume using a 3D ultrasound technique, which allows reliable measurements in a relatively short time. With the inclusion of patients suffering from cardiovascular diseases, we extend previous reports and show that this approach appears to be practical not only in preclinical atherosclerosis. Furthermore, we were able to demonstrate the individual role of CVRF in a multivariate model.

### 4.2. Limitations

A limitation of the present study is that most of our patients were in the low to intermediate risk population. Therefore, the findings of this study are probably not transferable to a high-risk population. Furthermore, our sample size was too small to assess clinical events correlated to plaque volume. Consequently, the findings of the present study should be confirmed with larger studies in the future.

## 5. Conclusions

Measurement of plaque volume in the carotid and femoral arteries by 3D ultrasound appears to be a practical and reproducible technique which allows the exact determination of atherosclerotic plaque burden. We observed a significant association between CVRFs and cardiovascular diseases with atherosclerotic plaque burden. When comparing the influence of risk factors, smoking appeared to be primarily associated with total and femoral plaque burden, whereas hyperlipidaemia was more associated with carotid plaque burden. Further studies are needed to determine whether plaque volume determination by 3D ultrasound may have potential as an additional screening tool in risk stratification.

## Figures and Tables

**Table 1:** Characteristics of the study population.

	Total Population (n = 404)	Low Total Plaque Volume (n = 202, 50%, 0–255 mm^3^)	High Total Plaque Volume (n = 202, 50%, 256–2048 mm^3^)	*p* Value
**Age, years**	64 (56–71)	60 (54–67)	67 (60–74)	<0.001
**Female, n (%)**	174 (43.1)	104 (51.5)	70 (34.7)	0.001
**Body mass index, kg/m^2^**	25.4 (23.5–28.2)	25.3 (23.2–28.3)	25.8 (23.9–28.2)	0.483
**Hypertension, n (%)**	264 (65.3)	107 (53)	157 (77.7)	<0.001
**Family history for CV-disease, n (%)**	96 (23.8)	58 (28.7)	38 (18.8)	0.020
**Smoking (pack years)**	12 (± 18.5)	8.7 (± 14.9)	15.3 (±21)	0.010
**Hyperlipidaemia, n (%)**	354 (87.6)	175 (86.6)	179 (88.6)	0.546
**Diabetes mellitus, n (%)**	51 (12.6)	20 (9.9)	31 (15.3)	0.100
**Framingham risk score, (%)**	12.8 (7.5–21.6)	9.4 (5.3–16)	17.1 (9.8–26.3)	<0.001
**hs-CRP, mg/dL**	0.18 (0.09–0.37)	0.16 (0.08–0.35)	0.21 (0.09–0.42)	0.031
**Total cholesterol, mg/dL**	193 (164.3–225)	199 (173.3–232.5)	183.5 (158.3–213)	0.003
**LDL-cholesterol, mg/dL**	115 (91–146)	120 (97.3–150)	109 (86–138.8)	0.009
**HDL-cholesterol, mg/dL**	58 (47–73)	61 (48.3–76)	56 (45–67.8)	0.009
**Triglyceride, mg/dL**	129 (94–179.8)	122 (83.3–171.5)	135 (97.3–190.8)	0.030
**Creatinine, mg/dL**	0.94 (0.83–1.08)	0.91 (0.82-1.02)	0.98 (0.83–1.12)	0.003
**Lipid lowering therapy**	234 (58.1)	103 (51)	131 (64.9)	0.005
**Antihypertensive therapy**	238 (59.1)	100 (49.5)	138 (68.3)	<0.001
**Antidiabetic therapy**	47 (11.7)	18 (8.9)	29 (14.4)	0.088

Parameters are median (interquartile range) or mean (± standard deviation) as indicated for continuous variables or number (percentage) for categorical variables. CV-disease = cardiovascular disease, LDL = low density lipoprotein, HDL = high density lipoprotein, hs-CRP = high-sensitive C-reactive protein.

**Table 2:** Distribution of the total plaque volume divided in low and high total plaque volume according to vascular diseases.

	Total Population (n = 404)	Low Total Plaque Volume (n = 202, 50%)	High Total Plaque Volume (n = 202, 50%)	*p* Value
**CAD, n (%)**	122 (30.2)	41 (20.3)	81 (40.1)	<0.001
**CVD, n (%)**	39 (9.7)	14 (6.9)	25 (12.4)	0.064
**PAD, n (%)**	32 (7.9)	5 (2.5)	27 (13.4)	<0.001
**Any vascular disease, n (%)**	155 (38.4)	53 (26.2)	102 (50.5)	<0.001
**No vascular disease, n (%)**	249 (61.6)	149 (59.8)	100 (40.2)	<0.001

Parameters are given as number (percentage). CAD = coronary artery disease, CVD = cerebrovascular disease, PAD = peripheral arterial occlusive disease. Vascular disease was defined as history of CAD, CVD, or PAD.

**Table 3:** Ankle-brachial index, pulse wave velocity, carotid and femoral IMT and carotid, femoral and total plaque volume in the total population and in patients with high and low total plaque volume.

	Total Population (n = 404)	Low Total Plaque Volume (n = 202, 50%)	High Total Plaque Volume (n = 202, 50%)	*p* Value
**Total plaque volume, mm** ^3^	255.5 (83.1–514)	83.3 (22–156.8)	513 (371.5–772.8)	<0.001
**Femoral plaque volume, mm** ^3^	139.5 (28.3–284)	39.5 (0–98.8)	283 (188–481.8)	<0.001
**Carotid plaque volume, mm** ^3^	79.5 (12.3–240.3)	19.5 (0–56.5)	239.5 (109–403.5)	<0.001
**Femoral IMT, mm**	0.48 (0.44–0.53)	0. 47 (0.42–0.52)	0.50 (0.45–0.54)	<0.001
**Carotid IMT, mm**	0.72 (0.62–0.82)	0.68 (0.60–0.78)	0.75 (0.68–0.85)	<0.001
**Ankle-brachial index**	0.91 (±0.10)	0.93 (±0.10)	0.89 (±0.10)	0.002
**Pulse wave velocity, m/s**	5.8 (4.8–7.1)	5.5 (4.7–6.8)	6.1 (4.8–7.4)	0.012

Parameters are median (interquartile range) or mean (± standard deviation) as indicated. IMT = intima media thickness.

**Table 4:** Distribution of total, femoral and carotid plaque volume depending on the number of cardiovascular risk factors (CVRF).

		Total Plaque Volume	Femoral Paque Volume	Carotid Plaque Volume
	n (%)	Median (IQR), mm^3^	Median (IQR), mm^3^	Median (IQR), mm^3^
**1 CVRF**	n = 123,31	**152 (58–360)**	**79 (14–226)**	**35 (5–160)**
**2 CVRF**	n = 187,47	**257 (83–506)**	**144 (29–290)**	**87 (11–222)**
**≥ 3 CVRF**	n = 88,22	**448 (167–701)**	**215 (84–445)**	**139 (52–351)**

Statistical significant differences (*p* < 0.05) between 1 CVRF, 2 CVRFs and ≥3 CVRFs are shown in bold. IQR = interquartile range.

**Table 5:** Distribution of total and femoral plaque volume depending on smoking behaviour.

	Total Plaque Volume	Femoral Plaque Volume	Carotid Plaque Burden
	Median (IQR), mm^3^	Median (IQR), mm^3^	Median (IQR), mm^3^
**Smokers (n = 102)**	**362 (91–666)**	**196 (55–396)**	73 (12–283)
**Non-smokers (n = 302)**	**240 (72–496)**	**124 (21–253)**	81 (13–223)
**<30 pack-years (n = 119)**	**226 (50–522)**	**125 (15–298)**	63 (5–250)
**31–60 pack-years (n = 55)**	**393 (145–672)**	**234 (103–420)**	120 (27–275)
**>60 pack-years (n = 6)**	**706 (333–1079)**	**443 (164–819)**	195 (110–402)

Statistical significant differences (*p* < 0.05) between smokers and non-smokers as well between individuals having smoked <30, 31–60 or >60 pack-years are shown in bold. IQR = interquartile range.

**Table 6:** Multivariate prediction model for total, femoral and carotid plaque volume.

	High Total Plaque Volume		High Femoral Plaque Volume		High Carotid Plaque Volume	
**Multivariate proportional odds**	OR (95CI)	*p* value	OR (95CI)	*p* value	OR (95CI)	*p* value
**Smoking**	**2.68 (1.52–4.71)**	**0.001**	**2.69(1.57–4.62)**	**<0.001**	1.50 (0.86–2.63)	0.154
**Hypertension**	**1.95 (1.17–3.25)**	**0.010**	**1.91 (1.17–3.11)**	**0.010**	**1.89 (1.12–3.19)**	**0.016**
**Hyperlipidaemia**	1.02 (0.52–2.01)	0.945	0.95 (0.50–1.83)	0.886	**2.23 (1.09–4.55)**	**0.028**
**Diabetes**	1.21 (0.60–2.46)	0.594	0.70 (0.36–1.38)	0.306	1.34 (0.65–2.74)	0.428
**Family history of CV disease**	0.63 (0.37–1.09)	0.097	1.05 (0.63–1.75)	0.865	**0.45 (0.26–0.78)**	**0.005**
**Age**	**1.09 (1.06–1.12)**	**<0.001**	**1.07 (1.04–1.10)**	**<0.001**	**1.10 (1.06–1.13)**	**<0.001**
**PAD**	**4.33 (1.46–12.86)**	**0.008**	**5.06 (1.75–14.69)**	**0.003**	2.34 (0.88–6.21)	0.089
**CAD**	**1.87 (1.10–3.19)**	**0.020**	1.54 (0.93–2.55)	0.091	**2.19 (1.27–3.80)**	**0.005**
**CVD**	1.19 (0.53–2.70)	0.673	0.67 (0.31–1.44)	0.298	**2.93 (1.19–7.26)**	**0.020**
**Chronic kidney disease**	1.80 (0.91–3.55)	0.090	1.36 (0.71–2.57)	0.353	1.45 (0.74–2.84)	0.285

The odds ratios for increased total, femoral and carotid plaque volume are demonstrated. CV = cardiovascular, PAD = peripheral arterial occlusive disease, CAD = coronary artery disease, CVD = cerebrovascular disease, OR = odds ratio. Statistically significant odds ratios are shown in bold.
